# Serious games for health: three steps forwards

**DOI:** 10.1186/s41077-017-0036-3

**Published:** 2017-02-04

**Authors:** David Drummond, Alice Hadchouel, Antoine Tesnière

**Affiliations:** 1 Ilumens Simulation Department, Paris Descartes University, 45 rue des Saint Pères, 75006 Paris, France; 2 Pediatric Pulmonology, Necker-Enfants Malades Hospital, Assistance Publique-Hôpitaux de Paris, 149 rue de Sèvres, 75015 Paris, France; 3 Surgical Intensive Care Unit, Cochin Hospital, Assistance Publique-Hôpitaux de Paris, 27, rue du Faubourg-Saint-Jacques, 75014 Paris, France

**Keywords:** Video game, Health education, Patient education, Medical education

## Abstract

Serious games are educational tools which are more and more used in patient and health professional education. In this article, we discuss three main points that developers and educators need to address during the development of a serious game for health. We first explain how to develop motivating serious games by finding a point where the intrinsic and extrinsic motivations of end users can converge. Then, we propose to identify the features of serious games which enhance their learning effectiveness on the basis of a framework derived from cognitive science and called “the four pillars of learning.” Finally, we discuss issues and solutions related to the evaluation of serious games.

## Background

New technologies have invaded our world, and while they can impair learning [1], they should also be considered as important tools to enhance learning in the context of the major shift occurring in education. For thousands of years, the limiting resource in education was the learning content: during the Middle Age in Western Europe, knowledge was contained in rare and precious books which were copied in monasteries, one at a time. Learning content began to be disseminated after the invention of the printing press by Gutenberg around 1450, but this access vastly increased by the World Wide Web and its search engines such as Google (1998). Today, when anyone can find any information on the Internet, learning content is no longer rare and precious, whereas the educational methods, i.e. the process of how knowledge is acquired, draw increasing attention. Information corresponds to facts provided about something or someone. By contrast, knowledge acquisition is the process of extracting, structuring, and organizing information. It requires time and corresponds to the theoretical or practical understanding of a subject, through education, experience, or reflection.

Serious games are potentially interesting tools to acquire knowledge, both for their motivational effect and the pedagogical principles they include such as a user-centred approach, interactivity, repetition, and continuous feedback [2]. They are attracting growing attention in the health area, with the development of large conferences dealing with games such as the “Games for Health” conferences in the USA (https://gamesforhealth.org/) and in Europe (http://www.gamesforhealtheurope.org/). The development of serious games is complex because of their twofold objective, being both motivating and educational. In this article, we discuss three main points that developers and educators need to address during the development of a serious game. We first explain how to develop motivating serious games by finding a point where the intrinsic and extrinsic motivations of the end users can converge. Then, we propose to identify the features of serious games which enhance their learning effectiveness on the basis of a framework derived from cognitive science and called “the four pillars of learning.” Finally, we discuss issues and solutions related to the evaluation of serious games.

## The convergence of motivations

A major factor in education is the time allowed to the learning process, a longer time being associated with better learning outcomes [3, 4]. Because video game players spend many hours per week on their computer, video games were considered as an interesting educational method [5]. By incorporating some learning content into video games, it was thought that players using these “serious” games would still enjoy the game while learning for hours [6]. This proposal led to important disappointments about both the enjoyment that serious games may provide and their learning effectiveness. *Arden: The World of Shakespeare* is a classical illustration of such a failure. This project was intended to be a massively multiplayer online learning game for teaching undergraduate learners the works of William Shakespeare. It was designed to be a plug-in for the off-the-shelf game *Neverwinter Nights.* When the game was launched, the virtual world turned out to be a great looking but players were dissatisfied with the gameplay and abandoned the game. This led the MacArthur Foundation which initially founded the game to pull out of this ambitious project [7].

Thus, developing a motivating serious game requires the developer to be interested in the motivation of the end users. Following the self-determination theory of Deci and Ryan, three types of learners can be defined: the amotivated, the extrinsically motivated, and the intrinsically motivated learners [8]. The amotivated learners do not value the activity of learning and do not believe that it will yield a desirable outcome. When asked to use a serious game, the only aspect they enjoy is the “sensory delight” related to the graphical environment and sound effects used in the game, known in the gaming business as “Eye Candy” [6]. Once this transient enjoyment fades, they quickly abandon the serious game. The extrinsically motivated learners do not value the activity of learning but do believe that it can yield a desirable outcome, for example obtaining a high examination score at the end of the training. Their difficulty is to find the energy to learn in order to achieve a future, desirable outcome. Finally, intrinsically motivated learners enjoy the activity of learning itself and/or are interested in the subject matter: from their perspective, the serious game represents one opportunity among many others (conferences, textbooks, etc.) to learn.

Therefore, in our opinion, for both amotivated and intrinsically motivated learners, serious games have little or no motivational value compared to conventional instruction methods, because these tools represent the same kind of constraint for the former and the same kind of opportunity to learn for the latter. This may explain why a meta-analysis of the motivating effect of serious games found that serious games were not more motivating than conventional instruction methods [9].

**Fig. 1:**
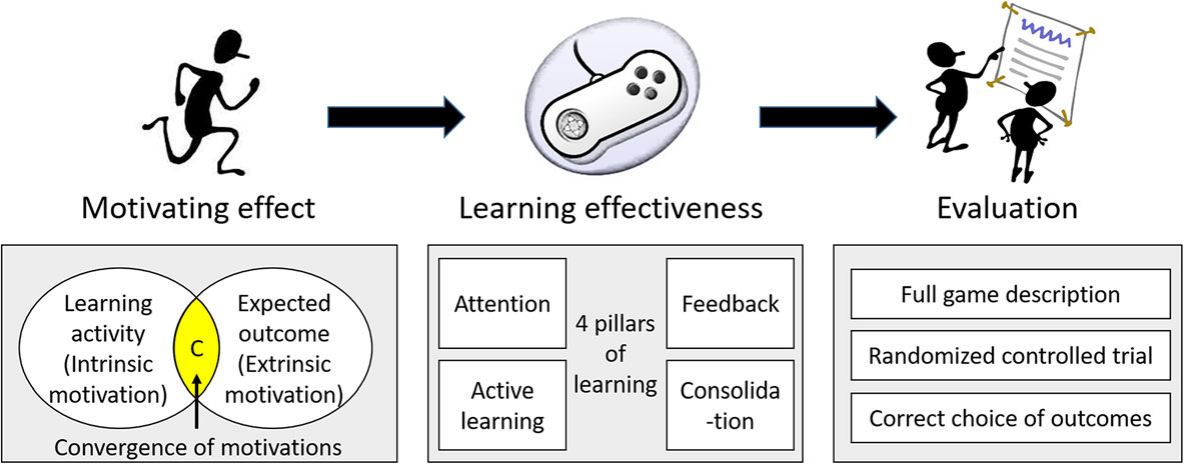
The three main points to discuss during the development of a serious game. Firstly, users need to be motivated to play the serious game in order to access the learning content. Motivating serious games integrate the extrinsic motivation of the player for a specific outcome (e.g. practicing as a medical doctor) into the learning activity (e.g. a simulation game on the management of cardiac arrest), making this learning activity desirable (apparition of intrinsic motivation for the learning activity). This phenomenon was called “the convergence of motivations.” Secondly, the learning potential of serious games should be maximized. The “four pillars of learning,” a framework derived from cognitive science finding [12], may be useful for developers and educators to enhance the learning effectiveness of their games. Finally, serious game should be evaluated in order to progress towards evidence-based education

However, we think that serious games may be interesting for extrinsically motivated learners. These learners consider the learning process as a painful but necessary step to reach a desirable, enjoyable outcome. For this category of learners, serious games allow them to experiment this enjoyable outcome virtually, in advance, while they are learning. In other words, serious games combine the enjoyment of this future outcome made virtually present with the learning activity (Fig. 1).

This combination was present in a very successful serious game developed in the health area, the *Re-Mission* game. In this game developed for young cancer patients, players controlled a humanoid nanorobot which had the mission to destroy different types of cancer at a cellular level, while learning the importance of compliance to chemotherapy. Most of these young cancer patients were likely to be extrinsically motivated learners, with a profound hope to reach a very desirable outcome, being rid of their cancer, although not enjoying learning about the importance of being compliant with their chemotherapy. The strength of the game was to make this desirable outcome virtually present, by allowing players to destroy several cancers, while delivering important messages about compliance to chemotherapy. The serious game could preserve both its enjoyment, as attested by the more than 200,000 copies delivered, and its learning effectiveness, demonstrated in a randomized controlled trial involving 375 patients which revealed that playing this game was associated with better knowledge, self-efficacy, and adherence to oral chemotherapies [10].

By contrast, there are several examples of serious games that failed to be motivating because their developers thought that putting algebra problems in a 3D virtual world or placing the periodic table of the elements in a shooting arcade would motivate students to play while learning [7]. In these situations, which did not involve any desirable outcome, students considered serious games as poorly motivating as their traditional lectures.

Combining the motivation for the activity of learning itself (intrinsic motivation) with the motivation for a future desirable outcome (extrinsic motivation) is thus essential to develop motivating serious games (Fig. 1). It may explain the enthusiasm of medical students for simulation games in which they play the role of medical doctors [11]. The association of their final objective—practicing as a doctor—made virtually present and possible, with a learning activity—making decisions to save the life of a virtual patient—is promising to motivate these students to learn.

Developers of games in the health area should consider this “convergence of motivations” if they want their product to be motivating from the perspective of their learners. However, even a serious game played for hours can fail to reach its educational purpose if developers neglect to consider the key instructional features which will allow the learning process and which are developed below.

## Learning benefits expected from serious games: insights from neurosciences

**Fig. 2:**
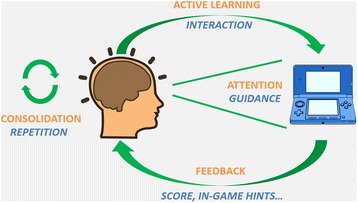
Pillars of learning (in *orange*) and the value of video games (in *blue*)

Cognitive science has identified four main pillars of learning: attention, active learning, feedback, and consolidation [12] (Figs. 1 and 2). These four pillars need to be carefully included in serious games (Table 1) and are addressed individually in the next four sections.

**Table 1:** The four pillars of learning, a new framework to enhance the learning effectiveness of serious games

Pillars of learning	Objective	Practical implications for serious games development	Practical implications for serious game implementation	Future directions of research
Attention - Alerting network	Reaching medium level of arousal	None	None	Links between serious game design, level of arousal, learning effectiveness
Attention - Orienting network	Selecting relevant information	Promote strategies that help to select relevant information (modelling/examples, modality/use of the audio channel for verbal explanations to guide visual search, feedback, integration of relevant information in virtual tools)	Educators should discuss with learners at the end of the serious gaming session in order to ensure that they identified the relevant information	New strategies integrated in games for guiding the player towards relevant information
Attention - Executive network	Staying focused on the serious game	None	Because learners are not always immersed in the game although they do learn, the environment shall stay clear of distractions during the serious gaming session	Differences between immersion, engagement, and repercussions on learning effectiveness
Active learning	Being engaged during learning	Promote interactivity rather than convey the learning content via text or audio explanation	None	Description of game features that enhance interactivity
Feedback	Evaluating the gap between the objective and the actual performance	Promote the use of feedback which deals with the task completed, not with the self-esteem	Educators can debrief the performance of players at the end of the serious gaming session	Exploration of forms of feedback which are the most effective in serious gaming
Consolidation	Achieving long-term memory	Promote the repetition of interactions with important learning content inside the game	Promote spaced-education with multiple training sessions and various educational methods	Description of forgetting curves after different types of serious gaming and determination on how often refreshers should be proposed

### Attention

Neuroscientists consider that attention consists of three attentional networks: the alerting network, the orienting network, and the executive network [13, 14]. These three networks are essential in the learning process.

Firstly, learners should stay alert during the learning process. The effects of alertness on memory seem to follow an inverted U-shaped relationship, with medium level of arousal associated with the highest gains in knowledge [15]. In serious games, the level of alertness is the result of three components: the graphic and sound environment of the game, the challenge proposed, and the motivation of the player. Technological advancements on video games’ effects, including the progress in video game graphics and the increased structural complexity of sound effects, were shown to be associated with higher levels of arousal in action video games [16, 17]. The level of challenge proposed by the game was also shown to be a key feature of the engagement of the player, with positive effects of learning [18]. This challenge should be in line with the skills of the learner because a high challenge with low skills may lead to frustration whereas a low challenge with high skills may lead to boredom [19]. Finally, the motivation of the player is also essential to reach sustained vigilance while playing, because motivation and arousal are closely linked [20].

The second network of attention (orienting network) can be described as a filter which selects some information from the environment at the expense of other information. There is evidence that guided instruction, which provides learners with direct instructional guidance on the concepts and procedures required by a particular discipline, leads to superior learning outcomes than unguided or minimally guided environment, in which learners must discover or construct essential information for themselves [21]. This highlights the importance for the instructor (or the educational tool) to help the learner to select the relevant information. A meta-analytic review of the role of instructional support in serious games confirmed that the use of instructional support was associated with better learning outcomes [22]. Among different categories of instructional support, facilitating learners in selecting relevant information was the most effective solution. Three types of support related to the selection of relevant information were studied: modelling (showing which information is important or how to solve a problem), modality (the use of the audio channel for verbal explanations to guide visual search), and feedback (which allows players to know if the information is relevant or not). All these types of instructional support were associated with improved learning outcomes. A fourth way to guide the player to select the relevant information is to integrate this information in the virtual tools that the player needs to use to reach the objective of the game. For example, in the Re-Mission game, players needed to search for chemotherapy to load their weapons in order to destroy the cancer cells. Players were orientated continuously towards chemotherapy during the game and thus learnt implicitly that adherence to chemotherapy was the key to cure cancer [10]. Developers should consider such strategies to help their end users to learn efficiently.

Executive control is the third attentional network. It allows one to concentrate on the task without being distracted. The ability to maintain sustained attention on a task without being distracted by the environment or his own thoughts is essential for learning. In serious games, it was originally thought that the immersion experienced during the game would prevent users from being distracted. Indeed, immersion is defined as “the sensation of being surrounded by a completely other reality […] that takes over all of our attention, our whole perceptual apparatus” [23]. Jennet et al. explained that “immersion involves a lack of awareness of time, a loss of awareness of the real world, involvement and a sense of being in the task environment” [24]. However, if immersion allows learners to stay focused on the game, emerging evidence suggests that high levels of immersion may allow them to master the game, but not to achieve the learning outcomes [25]. An explanation may be that high levels of immersion are associated with episodic memory, corresponding to the memory of the context (times, places, associated emotions, etc.) whereas low levels of immersion allow an appropriate distance for the creation of declarative memory, which is the memory of facts and abstract concepts [26]. Thus, when the learning objectives are more complex than simple retention and require a deep understanding of concepts, lower levels of immersion should be preferred. This implies that the environment in which a serious game is played should be clear of other sources of distraction, because the users who are learning abstract concepts during serious gaming sessions are not necessarily immersed and need to use their inhibitory control to stay focused on the task.

### Active learning

Active learning engages students in the process of learning through activities, as opposed to passively listening to an expert [27]. Active learning has been shown to be more effective than traditional didactic lecture, for both knowledge acquisition and behavioural change [27–29]. The strength of serious games is that they encourage active learning because they are interactive by essence. A serious game cannot be completed without the intervention of its user, as opposed to a lecture which will continue whether or not the learner is listening to it. A meta-analysis of the learning effectiveness of simulation games found that the games which actively engaged trainees in learning were more effective than those which passively conveyed the instructional material via text or audio explanation [30]. The game *Staying Alive* (http://www.stayingalive.fr/index_us.html) which was designed to teach the management of a cardiac arrest to the general public is a good illustration because it involves different levels of interactivity. In a first part, the explanations on the recognition of a cardiac arrest are limited to text boxes which require the player to click to the next one. By contrast, when the player has to provide virtual chest compressions, he is asked to choose his hand position between different proposals and to click repeatedly with the correct time interval to deliver compressions with the correct rate. In a study still ongoing in our simulation centre (ClinicalTrials.gov registry number 02758119), it appears that second year medical students who were pre-trained with the serious game and assessed on a physical manikin were more likely to provide chest compressions with a correct hand position and rate, whereas no progress was observed regarding the recognition of the cardiac arrest. Thus, future serious games should not be limited to a self-paced environment in which the player simply click to move to the next text box containing the relevant information but rather be fully interactive by integrating this information in actions that the player need to perform. In order to maximize the learning effectiveness of the serious game, these actions need to be similar to those performed in the “real world.”

### Feedback

According to the *temporal difference learning* theory, the brain can be seen as a machine that generates predictions and verifies them to improve its next prediction [31]. Learning occurs by minimizing the difference between the expected and actual outcome, through the feedback received [12]. Increasing evidence suggests that computations described by temporal difference learning theory are actually performed in the human brain [32, 33]. Feedback is therefore essential for learning, and it was believed that “the positive effects of feedback interventions on performance has become one of the most accepted principles in psychology” [34]. However, a comprehensive meta-analysis of laboratory experiments suggested that while feedback improved performance by 0.4 of a standard deviation on average, it reduced performance in over one third of the experiments [35]. In searching for the moderators, the authors found that feedback effectiveness decreased when the feedback dealt more with the learner than with the task. For example, feedback interventions in the form of a praise or a blame were less effective than feedback which focused on the task. This same meta-analysis found that computerized feedback interventions, which are likely to focus attention on the task, were associated with higher effectiveness compared to verbal feedback interventions.

In serious games, feedback can be integrated in different forms: progress bars, scoring, achievements, experience points, virtual currencies, etc. [36]. The player can perceive the difference between the performance expected in the serious game and his actual performance through the feedback received. More research is needed to define the forms of feedback which are the most effective in serious gaming. In cognitive training games, there is emerging evidence that the different types of feedback are not equally effective. It was shown that the inclusion of real-time scoring during play negatively impacted training improvements of the participants [37]. The authors suggested that real-time scoring distracted the learners from the core cognitive training task, which may also happen in serious games. Therefore, while feedback is essential for learning, the way it should be integrated in serious games deserves further investigation.

### Consolidation

The memory consolidation hypothesis Müller and Pilzecker described 100 years ago continues to guide memory research [38]. Processes that allow memory to consolidate have become increasingly well understood [39]. The first stages of the acquisition of a skill, such as reading or driving, require the learner to stay very concentrated, because these processes are conscious. Repeated trainings allow the brain to shift to faster and unconscious networks [40]. Neuroimaging revealed that during acquisition of a motor skill, there is a shift from prefrontal regions of the cortex to the premotor, posterior parietal, and cerebellar cortex structures [41]. In the field of serious games, learners learn more when multiple training sessions are involved [9] to allow these shifts in neural network utilization to occur.

This repetition is even more effective if the learner alternates training sessions and rest periods. Reinforcement using “spaced training,” where training sessions alternate with rest periods, leads to longer lasting memories than massed training [42, 43] and better transfer to real situations [44]. On the basis of these findings, “spaced education” in the medical field has been shown in randomized trials to improve knowledge acquisition, boost learning retention for up to 2 years, and durably improve clinical behaviour [45–47]. Spaced education can be integrated in serious games: an online spaced-education game to teach and assess medical students was shown to be effective in improving and assessing students’ knowledge [48].

Finally, this repetition is more effective if it involves different educational methods. A meta-analysis on the learning effectiveness of simulation games, a subgroup of serious games, found that they should be used as a supplement to lecture, discussion, tutorials, or other instructional methods in order to maximize their learning potential [30].

In summary, the development of serious games may benefit from scientists’ growing understanding of the human brain and of the mechanisms involved in learning. Then, the learning effectiveness of serious games should be evaluated rigorously through a scientific evaluation that we propose next.

## Evaluating serious games: towards evidence-based education

In medicine, the best evidence of the effectiveness of an intervention is the result of randomized controlled trials (RCTs) and of systematic reviews of these studies. It should be the same in the educational field [49, 50]. However, serious games on the market are so diverse in their content, objectives, and users and that they preclude high-quality meta-analyses [51]. Several authors have tried to propose a classification system for serious games to form more homogeneous groups, but a universal classification system has failed to be adopted [52–54], possibly because research in serious games involves authors with different backgrounds. Graafland and colleagues recently provided the first consensus-based framework for the assessment of specific medical serious games to address this problem [55]. Features linked to game description, rationale, functionality, validity, and data protection must be reported [55]. This framework allows educators and game designers to compare and validate new serious games in a consistent way.

Furthermore, the study designs of the articles evaluating serious games are very diverse. Less than 20% are RCTs [56]. Choosing an appropriate control group and a relevant outcome are the main difficulties encountered in RCTs by researchers.

Ideally, RCTs evaluating serious games should include two control groups, one group receiving no education and the other receiving another form of education [57]. The use of a control group who receives no education is essential to demonstrate the effectiveness of a serious game and still remains ethical because it happened that an intervention group playing a serious game failed to reach higher levels of knowledge and/or skills than a control group receiving no education [58]. The use of a second control group receiving another form of education is also important. Previous research in simulation-based education showed that simulation technology was more effective than no education, but the difference was less convincing relative to other forms of education [59, 60]. In academic settings, serious games should be compared to another form of education to promote their use. By using these two control groups, it also becomes possible to evaluate whether a serious game can be a pragmatic solution between no education and a form of education that is more effective but not readily accessible to learners, such as long and expensive trainings in simulation centres.

The choice of outcome is also complicated in the serious game field. Four levels of outcome can be used to evaluate medical education, following the model of Kirkpatrick [61, 62]: satisfaction (level 1), acquisition of knowledge and skills (2b), retention of knowledge and skills over a period of time (2c), behavioural change (3), and patient outcomes (4).

All these outcomes are complementary and relevant for the evaluation of serious games but do not have the same value. Each step-up among these levels is associated with both (1) more convincing evidence on the value of the educational intervention and (2) increased difficulty to conduct the study and demonstrate a difference between the experimental and control groups. Although most studies assess users’ satisfaction, research on serious games should at least address the acquisition of knowledge and skills (level 2), because there is no evidence that satisfaction alone leads to effective learning. Knowledge can be assessed simply by questionnaires, while physical simulation using manikins represents a new, safe, and appropriate material to evaluate the acquisition of skills after playing a serious game.

Besides RCTs which are essential but limited to a binary answer (yes/no) about the effectiveness of a serious game, other study designs can offer valuable information on how to develop effective serious games. A new approach is represented by neuroscientific studies evaluating the impact of playing a serious game on specific neural circuits. For example, the Re-Mission videogame for cancer patients was shown to activate the reward-related mesolimbic neural circuits which are involved in the motivation to play [63]. The authors demonstrated that this activation was primarily the consequence of the gameplay of the Re-Mission game which involved a lot of interactivity rather than the consequence of the effects of vivid and dynamic sensory stimulation. Such studies can improve our understanding of the mechanisms by which serious games can be motivating and enhance learning.

## Conclusions

Serious games are attracting growing attention in the health area. Developing a successful serious game is complex, and we proposed cues to avoid two classical pitfalls. First, in order to obtain a serious game played by its end users, we recommended to find a point where the intrinsic and extrinsic motivation of the players can converge, i.e. where the players can enjoy a future desirable outcome made virtually present in the game. Second, developers should consider the four pillars of learning described to avoid the development of a game which does not fulfil its educational objectives. Researchers in the field should continue to explore how these four pillars of learning can be used to enhance serious game engagement and effectiveness. Finally, evaluation of serious games using a standardized framework will help to legitimize the enthusiasm observed in the health area for these tools.

## Abbreviation

RCT: Randomized controlled trial
